# ROBOT: A Tool for Automating Ontology Workflows

**DOI:** 10.1186/s12859-019-3002-3

**Published:** 2019-07-29

**Authors:** Rebecca C. Jackson, James P. Balhoff, Eric Douglass, Nomi L. Harris, Christopher J. Mungall, James A. Overton

**Affiliations:** 1 Knocean Inc., Toronto, Ontario Canada; 2 Renaissance Computing Institute, University of North Carolina, Chapel Hill, North Carolina USA; 3 Lawrence Berkeley National Laboratory, Berkeley, California USA

**Keywords:** Ontology development, Automation, Ontology release, Reasoning, Workflows, Quality control, Import management

## Abstract

**Background:**

Ontologies are invaluable in the life sciences, but building and maintaining ontologies often requires a challenging number of distinct tasks such as running automated reasoners and quality control checks, extracting dependencies and application-specific subsets, generating standard reports, and generating release files in multiple formats. Similar to more general software development, automation is the key to executing and managing these tasks effectively and to releasing more robust products in standard forms.

For ontologies using the Web Ontology Language (OWL), the OWL API Java library is the foundation for a range of software tools, including the Protégé ontology editor. In the Open Biological and Biomedical Ontologies (OBO) community, we recognized the need to package a wide range of low-level OWL API functionality into a library of common higher-level operations and to make those operations available as a command-line tool.

**Results:**

ROBOT (a recursive acronym for “ROBOT is an OBO Tool”) is an open source library and command-line tool for automating ontology development tasks. The library can be called from any programming language that runs on the Java Virtual Machine (JVM). Most usage is through the command-line tool, which runs on macOS, Linux, and Windows. ROBOT provides ontology processing commands for a variety of tasks, including commands for converting formats, running a reasoner, creating import modules, running reports, and various other tasks. These commands can be combined into larger workflows using a separate task execution system such as GNU Make, and workflows can be automatically executed within continuous integration systems.

**Conclusions:**

ROBOT supports automation of a wide range of ontology development tasks, focusing on OBO conventions. It packages common high-level ontology development functionality into a convenient library, and makes it easy to configure, combine, and execute individual tasks in comprehensive, automated workflows. This helps ontology developers to efficiently create, maintain, and release high-quality ontologies, so that they can spend more time focusing on development tasks. It also helps guarantee that released ontologies are free of certain types of logical errors and conform to standard quality control checks, increasing the overall robustness and efficiency of the ontology development lifecycle.

## Background

Ontologies are vital parts of the informatics ecosystem supporting life science research, enabling analysis of high-throughput datasets, data standardization and integration, search, and discovery. However, there is a lack of tools supporting the complete ontology development lifecycle, especially when compared with the software development lifecycle. This has resulted in many groups developing their own ad-hoc ontology development workflows, often with time-consuming and inefficient manual steps. In some cases, groups release ontologies without any kind of systematic workflow or quality control process, which can result in errors or problems with downstream applications or analyses.

Noy, Tudorache, Nyulas, and Musen (2010) describes a general ontology lifecycle with a focus on bio-ontologies [1]. First, requirements for the ontology are gathered. Then, the ontology is collaboratively developed in an ontology editor such as Protégé [2]. Once the requirements have been fulfilled, the ontology is published, and feedback is solicited from the community. Feedback is integrated back into development, and the ontology is continuously updated and released. At any point after the initial publication, the ontology may be deployed in other applications.

In broad strokes, this ontology development lifecycle reflects much of our experience of ontology development in the Open Biological and Biomedical Ontologies (OBO) community [3], circa 2010. A wide range of Semantic Web software exists to support these steps, including many tools for Web Ontology Language (OWL) ontology development. In practice, though, the OBO community has relied predominantly on the free and open source Protégé OWL editor for manual editing and conversion, and on a small set of other tools supporting OBO conventions.

Other than Protégé, the most prominent suite of tools used by the OBO community has been the Onto-animal suite developed by the He group [4] including Ontobee [5], Ontofox [6], and Ontorat [7]. These tools are free web services backed by a Virtuoso triplestore loaded with the latest version of all available OBO community ontologies, as well as some other ontologies. Ontobee is an ontology term browser. Ontofox implements the MIREOT term extraction method [8]. Ontorat implements template-based ontology term creation. Together with a few other tools, these support an extensible ontology development strategy [9] covering a range of ontology development tasks, many of which can combined and automated using a sequence of web-based API calls.

The core operations of the Onto-animal suite are driven by SPARQL queries against the centralized triplestore. This results in a number of limitations. First, only the specific version of each ontology loaded into that triplestore can be used. This is a particularly severe limitation during ontology development. Second, processing is done on the centralized server, limiting the processing power available to any user. Third, SPARQL has limited utility when working with OWL logical axioms.

These limitations are mitigated by running software locally, loading the desired versions of the desired ontologies, and using OWL API [10] for OWL-native processing. A number of tools used in the OBO community have done precisely this. We have seen a spectrum of development, from tools that are focused on a single project, to tools used by a dozen related projects, to the current push for tools that are shared across the OBO community.

Slimmer [11], created as part of the eNanoMapper ontology project [12], uses OWL API to create ontology subsets (also known as “slims”). A configuration file allows the user to specify which terms to include and which annotations to include on those terms. OntoPilot [13], developed for the Plant Phenotype Ontology, uses OWL API via Jython (a version of Python that runs on the Java Virtual Machine) to provide an integrated ontology development framework, including term imports, term creation, releases, and documentation.

The lack of automation seen circa 2010 led directly to a lack of standardization, with each ontology editor or group adopting a slightly different approach to manual editing in Protégé. This diversity of practices, even within the OBO community, made it a challenge to develop tools to serve multiple ontology projects. OWLTools [14] was designed for use by multiple OBO ontology projects, providing convenience methods on top of the OWL API. OWLTools includes the OBO Ontology Release Tool (OORT) [15], a command-line tool to release OWL- and OBO-format ontologies. OORT provides a series of basic commands to create a release pipeline for an ontology, including module extraction with MIREOT, support for multiple input ontologies, reasoning, and creation of ‘main’ and ‘simple’ release products.

ROBOT (a recursive acronym for “ROBOT is an OBO Tool”) was developed to replace OWLTools and OORT with a more modular and maintainable code base. It builds on previous experience to include a comprehensive set of automation capabilities to support an even wider range of OBO projects. Development began in 2015 and continues with more than 1000 commits from a dozen contributors. ROBOT is freely available open source software. Although we do not track our users, a recent GitHub search shows that at least 26 ontology projects in the OBO community have adopted ROBOT.

## Implementation

### Overview

ROBOT provides a standardized yet configurable way to support the ontology development lifecycle via a library of common high-level functionality and a command-line interface. ROBOT builds on OWL API and is compatible with all ontology syntaxes that OWL API supports: RDF/XML, OWL/XML, Turtle, OWL Functional Syntax, OWL Manchester Syntax, and OBO format. The source code is written in Java and is available from our GitHub repository [16] under an open source (BSD 3) license. It is also released as a Java library on Maven Central. ROBOT code can be used from any programming language that runs on the Java Virtual Machine (JVM). The command-line tool is packaged as a JAR file that can be run on Unix (including macOS and Linux), Windows, and other platforms supported by the JVM. This JAR file is available for download from the ROBOT GitHub site [16], along with platform-specific scripts for using ‘robot’ from the command line. Installation instructions and documentation are available from http://robot.obolibrary.org.

### Architecture

We previously described the basic architecture of the tool [17], which we summarize here.

The ROBOT source code consists of two parts: ‘robot-core’ and ‘robot-command’. ‘robot-core’ is a library supporting common ontology development tasks, which we call “operations”. ‘robot-command’ provides a command-line interface divided into “commands”, each of which wraps a ‘robot-core’ operation.

Most ROBOT operations package low-level functionality provided by OWL API into high-level functionality common to ontology development workflows in the OBO community. For best compatibility, we aim to match the exact version of OWL API used by ROBOT with the exact version used by the latest Protégé release. Some operations use Apache Jena [18]. Each operation works with Java objects that represent OWL ontologies, OWL reasoners, OWL classes, etc., while each command works with command-line option strings and files. The commands also perform various conversion and validation steps. The command-line interface uses the Apache Commons CLI library [19] for parsing commands.

Each operation has a set of unit tests built with JUnit [20] that are executed each time the final product (the JAR file) is generated. Each command in ROBOT is documented in its own web page (e.g. http://robot.obolibrary.org/reason). The web pages are authored in Markdown format and contain embedded command-line examples that are parsed and executed as part of our integration tests, with the results compared against a “gold standard” set of outputs. ROBOT’s ‘diff’ functionality is used when comparing ontology files, otherwise standard file comparison is used. This helps ensure correctness and consistency of documentation and code. The unit tests and integration tests are executed on any pull request onto the codebase via Travis continuous integration (Travis CI) [21], so that contributions to the codebase are verified.

### Commands and operations

ROBOT currently provides 15 operations (in the ‘robot-core’ library) and 19 commands (for the command-line interface). Some commands are quite specialized, and most ontology projects will not make use of all of them. Here we provide an overview of the most common and general commands. In each case, the core functionality is supported by operations in the ‘robot-core’ library, that can be used independently of the command-line interface from any programming language that runs on the JVM.

### Convert

A variety of OWL ontology formats are supported, including RDF/XML, Turtle, Manchester, OBO format, and more. To enable further interoperability, ROBOT includes a ‘convert’ command to change between supported ontology formats. A complete list of supported formats can be found in the ‘convert’ documentation [22].

### Reasoning

Reasoning is one of the most important operations in ROBOT. The ‘reason’ command covers two uses: *logical validation* of an ontology and *automatic classification*. In both cases, users can choose their preferred reasoner, which is used to perform inference. Large ontologies such as the Gene Ontology typically use ELK [23], which performs reasoning quickly using the OWL EL profile. Smaller ontologies with richer axiomatization, such as the Relations Ontology, typically use a complete OWL DL reasoner such as HermiT [24].

When the ‘reason’ command is invoked on an input ontology, ROBOT will initiate a reasoner using the OWL API Reasoner interface. The resulting inferences are checked to ensure the ontology is *logically coherent*: the ontology must be consistent and have no unsatisfiable classes (i.e., classes that cannot be instantiated without introducing an inconsistency). If the ontology is incoherent, this is reported and execution halts. ROBOT can optionally perform additional checks, such as ensuring that no two classes are inferred to be equivalent post-reasoning.

If the ontology is consistent, ROBOT will perform automatic classification. All direct inferred ‘subClassOf’ axioms are added to the ontology. Generation of other types of axioms can be configured.

The assertion of all inferred axioms is often a fundamental step in the release process for biomedical ontologies. Many of these ontology classes only assert a single named superclass (‘A subClassOf B’, where B is another class in the ontology), and zero or more anonymous superclasses and/or anonymous equivalent classes (‘A subClassOf/equivalentTo (R some B)’, where R is an object property). These anonymous classes allow the reasoner to make inferences, which are then asserted. Therefore, in the release version of an ontology, a class may have more than one named superclass.

The ‘reason’ command has additional “helper” commands. The ‘relax’ command asserts entailed subClassOf axioms according to a simple structural rule: an expression ‘A equivalentTo (R some B) and …’ entails ‘A subClassOf R some B’. This can be useful as consumers of bio-ontologies often expect to navigate these expressions, e.g., partonomy in GO and Uberon. The ‘relax’ command relieves the ontology developer from the need to assert these in addition to the equivalence axioms, and as such it is also often included in release workflows. Finally, the ‘reduce’ command removes redundant subClassOf axioms, and can be used after ‘relax’ to remove duplicate axioms that were asserted in that step.

The ‘materialize’ command uses an Expression Materializing Reasoner (EMR) to assert inferred expressions of the form ‘A subClassOf R some B’ [25]. Where the ‘reason’ command asserts inferred *named* superclasses, ‘materialize’ asserts *anonymous* superclasses. This is not part of the standard release cycle but can be beneficial for creating complete ontology subsets.

### Working with external ontologies

The OBO Foundry aims to coordinate ontologies in a modular fashion, such that parts of some ontologies can be used as building blocks for other ontologies. For example, the ChEBI chemical entities ontology [26] is used to construct OWL definitions for metabolic processes and activities in the Gene Ontology [27]. There are a variety of different strategies for leveraging external ontologies and managing dependencies between ontologies, depending on the use case.

### Extract

The ‘extract’ command creates a module based on a set of entities to extract (the “seed”). There are four different extraction methods (as specified by the ‘--method’ option): MIREOT, TOP, BOT, and STAR.

ROBOT’s MIREOT extraction method is based on the principle of the same name [8] and requires that one or more “bottom” entities are specified. Optionally, one or more “top” entities can also be specified. The command extracts all the “bottom” level entities and their ancestors up to the “top” level from the input ontology. If no “top” entities are provided, ancestors up to the top-level entity (‘owl: Thing’) are included.

The TOP, BOT, and STAR methods make use of the OWL API Syntactic Locality Module Extraction (SLME) implementation, which is guaranteed to capture all information logically relevant to the seed set [28]. The BOT method (“bottom”) includes all relationships between the input entities and their ancestors. The TOP method includes all relationships between the input entities and their descendants. Finally, the STAR method only includes all relationships between input entities. The STAR method produces the smallest outputs, while the TOP method typically produces the largest outputs.

In order to support ontology term provenance, the ‘extract’ command has an ‘--annotate-with-source true’ option that will annotate each extracted term with the URL of the source ontology that it is extracted from.

### Remove and filter

The ‘remove’ and ‘filter’ commands are used for fine-grained operations on OWL axioms. ‘remove’ allows users to choose which sets of axioms they wish to remove from a target ontology. ‘filter’ does the opposite, so that only selected axioms are copied from the input into a new output ontology.

These two commands work by starting with a seed set of entities, then applying various selectors to find related entities, and finally selecting which axiom types to remove or filter. We expect only a small number of “power users” to use this feature directly, but these commands will eventually provide a foundation for other higher-level commands.

These commands can be used to generate ontology subsets based on annotations by either filtering for or removing entities with the specified annotation. OBO Foundry ontologies often annotate classes with the ‘in subset’ property to specify where a class might be used. The annotation selector allows a user to provide a full annotation value or a pattern to match using regular expression.

### Merge

The ‘merge’ command combines two or more separate input ontologies into a single ontology. It also provides the ability to merge all imported ontologies of a single input ontology into one main ontology, which is often used when creating a release.

Merging imported ontologies (specified by import statements) into the input ontology is performed automatically, so that the user does not need to list each imported ontology as an input. We offer the option (‘--collapse-import-closure false’) to turn this feature off, supporting cases in which users may merge multiple input ontologies that have import statements but want to keep their imports separate.

### Querying and reporting

Ontology workflows typically include query operations over the ontology, producing reports which may be informative for both editors and users of the ontology -- for example, a table of all classes plus their textual definitions. Query operations can also be used for validation checks. The SPARQL query language provides a universal and declarative way for ontology maintainers to create ontology reports and validation checks [29]. ROBOT provides a convenient way to perform queries with the ‘query’ command, or validation checks using ‘verify’. Additionally, the ‘report’ command includes a configurable package of standard queries for OBO projects that can be used in any ontology workflow, without requiring the maintainer to be familiar with SPARQL.

### Query

ROBOT’s ‘query’ command runs SPARQL queries on ontologies or other RDF resources. This can be used by an ontology maintainer to either perform interactive queries, or more typically to include standard queries into an ontology workflow. The ‘query’ command wraps one of the few operations that uses Apache Jena [18], rather than OWL API. The Jena API allows ROBOT to load an ontology as a collection of triples contained by an RDF Model object. It provides a SPARQL query engine for those models, which we use to run all queries.

‘SPARQL SELECT’ queries produce a comma- or tab-separated table of results. ASK queries produce a file with a Boolean value. ‘SPARQL CONSTRUCT’ queries produce an RDF file, which can be further processed by ROBOT or merged back into the loaded ontology. ‘CONSTRUCT’s provide a convenient way of performing “macro” style expansion [30]. ‘SPARQL UPDATE’ queries insert and/or remove data directly in an ontology (as an RDF Model). ROBOT converts the updated RDF Model back to an OWL API ontology object to be saved in any of the supported syntaxes.

The ‘query’ command supports an option to load imported ontologies as named graphs with the ‘--use-graphs’ option. If this is set to ‘true’, the imports can be queried as named graphs (the name being that ontology’s IRI) and the default graph is a union of all graphs. Using the default graph is similar to conducting a ‘merge’ of all the imports prior to querying, but the distinction between imports would be lost in a ‘merge’.

### Verify

The ‘verify’ command is a variation on the ‘SPARQL SELECT’ execution. The queries are used to ensure that an ontology conforms to a predetermined set of conditions; for example, ensuring that no class has multiple textual definitions. Given a SELECT query, ‘verify’ will succeed (i.e., exit with status code 0) if no results are returned. It will fail (i.e., exit with a non-zero status code) if any results are return from the query. So, given a SPARQL query that SELECTs for invalid data, the ‘verify’ command will verify that the ontology (or other resource) does not contain any such invalid data.

### Report

The ‘report’ command is an extension of ‘query’ and ‘verify’ that provides a series of configurable quality control (QC) checks for an ontology and returns a spreadsheet or YAML output of the violations. The spreadsheet is output in either comma- or tab-separated format and easy for a user to read, while the YAML output can be easily parsed by other programs.

The QC checks include annotation checks, logical checks, and metadata checks. Annotations are important to facilitate human comprehension, so the ‘report’ command finds cases where missing or duplicate annotations could cause problems. Logical checks look at the structural coherency and consistency of the ontology. Finally, ‘report’ identifies missing ontology metadata, as specified by OBO Foundry recommendations.

There are three levels of violations that are reported: ERROR, WARN, and INFO. An ERROR is the most severe, such as a missing or duplicate label. By default, the ‘report’ command fails if there are any ERROR-level violations, halting any automated build processes. These types of violations must be fixed before publishing an ontology. WARN-level violations should be fixed as soon as possible, e.g. inferred one-to-one class equivalencies, which are typically unintended in OBO projects. INFO is for recommended fixes that help maintain consistency across OBO Foundry ontologies, such as beginning a definition with an uppercase letter and ending with a period. ‘report’ can be configured with a command-line option to fail on a different violation level or to never fail, regardless of any violations. We document each QC check with a suggestion for a manual fix that the user can apply.

A default “profile” with report levels for each QC check is provided by ROBOT, but users are also able to create their own profiles. In these profiles, they can change the violation levels of individual checks, choose to exclude certain checks, and add their own checks as SPARQL queries. For example, some ontologies may categorize a class lacking a textual definition as an error, while others may categorize this as a warning. One of our goals is to converge on a standard profile that is maximally useful for the set of all ontologies in the OBO library, encouraging adoption of common quality control checks.

### Repair

Although most problems raised by ‘validate’ and ‘report’ must be fixed manually, ROBOT also provides a ‘repair’ command that can automatically fix certain problems. The current implementation will merge annotations on duplicate axioms and update references to deprecated classes when they are annotated with a suggested replacement. We intend to extend ‘repair’ to a wider range of common problems for which the correct fix is clear.

### Templated ontology development

ROBOT provides a template-driven ontology term generation system. Users also have the option to plug in their own term generation system into their workflow, such as Dead Simple OWL Design Patterns (DOS-DPs) [31].

A huge amount of data is stored in spreadsheets and databases, and tabular formats are well suited to many sorts of data. ROBOT’s ‘template’ command allows users to convert tabular data into RDF/OWL format. A ROBOT template is simply a tab-separated values (TSV) or comma-separated values (CSV) file with some special conventions, which are outlined in the ROBOT ‘template’ documentation [32].

These templates can be used for modular ontology development. The template spreadsheets may be maintained as part of the ontology’s source code repository, and instead of directly editing the ontology file, developers edit rows in the spreadsheet which correspond to terms in the ontology. The ‘template’ command is then used to generate a module of the ontology, which is included as an import statement in the editors’ version of the ontology and merged during the release process.

### Workflows

A workflow consists of a set of tasks coordinated by some workflow system. Ontology workflows consist of tasks such as executing QC checks, building import modules, reasoning over ontologies, and generating various ontology release products.

ROBOT itself is not a workflow manager, although it allows multiple commands to be chained together into one long command. When chaining ROBOT commands, the output ontology from one command is passed directly as the input to the next command. For example, chaining may be used to replace two commands that merge ontologies and then reason over the merged product:

`robot merge --input ont-1.owl --input ont-2.owl --output merged.owl.

robot reason --input merged.owl --output reasoned.owl`.

Instead of creating the merged product and running ‘reason’ over that, it can be done in one command:

`robot merge --input ont-1.owl --input ont-2.owl reason --output reasoned.owl`.

The key advantage to chaining is that ontologies do not have to be serialized and parsed between each step; the same OWL API ontology object is maintained in memory and passed through the chain of ROBOT commands. For large ontologies, chaining can vastly improve ROBOT’s performance.

Because ROBOT commands can be executed on the command line, a number of different workflow systems can be used. We highlight the use of GNU Make [33], which is typically used to compile software. A Makefile consists of a set of rules used to make “targets”. In ontology development, the Makefile is used for automated tasks, such as preparing an ontology for release. In this case, the targets are usually ontology files. The “recipes” for the rules are Unix-style system commands, carried out by the ‘make’ command.

**Fig. 1:**
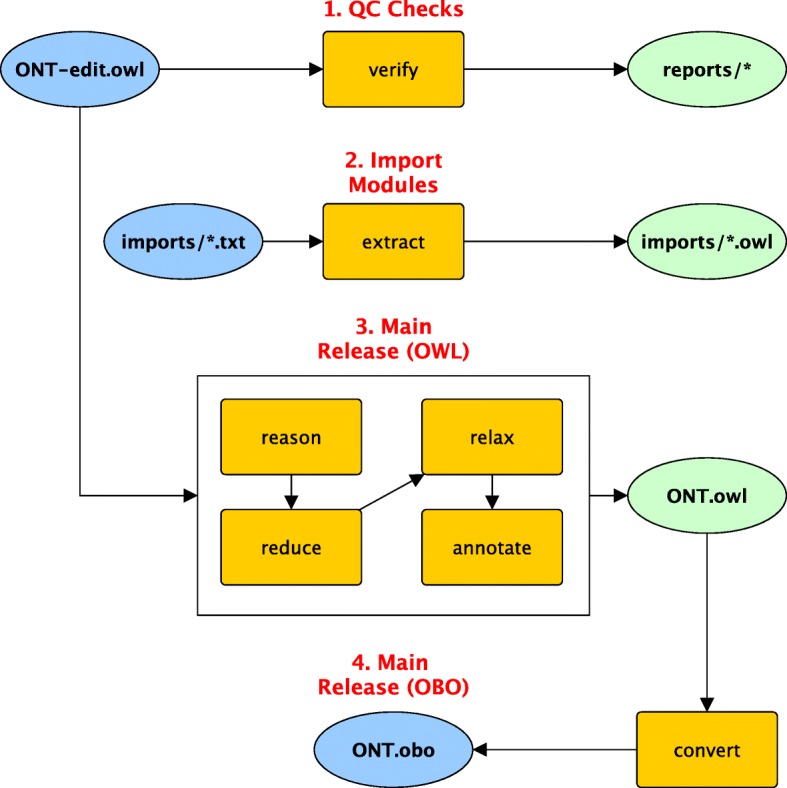
The ROBOT release workflow. A typical release workflow using ROBOT. The ontology edit file ONT-edit.owl is first verified as a quality control check with ROBOT ‘verify’. Then, text files containing lists of external ontology terms in the imports directory are used to regenerate import modules using ‘extract’, ensuring that the imports are up-to-date. ONT-edit.owl is then passed through a series of ROBOT commands (‘reason’, ‘relax’, ‘reduce’, and ‘annotate’) to generate the release, ONT.owl. Finally, ONT.owl is converted to OBO format

ROBOT commands can be used as the “recipes” to make the “targets”. A typical workflow will not use all 19 of the ROBOT commands. For example, not all ontology projects may use ROBOT templates and therefore not all release workflows need to include the ‘template’ command. Ontology developers can decide which commands are needed to perform the release and build a workflow around those commands. Figure 1 shows a standard way in which a selection of ROBOT commands is combined for a release workflow.

First, quality control checks are run over the editors’ version of the ontology with ‘report’ or ‘verify’. These look for equivalent classes, trailing whitespace in annotations, self-references, incorrect cross-reference syntax, and missing labels. The results are saved to a specified ‘reports/’ directory. If there are any ERROR-level violations, the task will fail and write the violations to a table so that they can be easily identified. This step allows developers to quickly see if new changes have introduced any problems within the ontology and fix them before releasing.

Assuming the initial QC check step has completed successfully, the next step is to create the import modules. The ROBOT ‘extract’ is run for each entry in a list of imports, which have corresponding term files (for the seed set) in the ‘imports/’ directory. This creates all the import modules in the same ‘imports/’ directory. This ensures that when an ontology is released with external terms, all external terms are up-to-date with the released versions of the source ontologies. Releasing out-of-date external terms can cause confusion, as the term will show both the old and new details in ontology search services like Ontobee [5] and the Ontology Lookup Service [34]. Additional QC checks can be run over the full ontology with imports using the ‘verify’ command or by running ‘report’ again.

Last, the main release products are created: the OWL file and the OBO file. To create the OWL release, the editors’ file is passed through a series of chained ROBOT commands: ‘reason’, ‘relax’, ‘reduce’, and ‘annotate’. This series of commands helps to ensure that the released ontology is both easy to browse and understand, as well as free of any redundant axioms. If any of these commands fail, the Make process will terminate with the corresponding error message. For example, if an ontology is incoherent the ‘reason’ step will fail. Finally, the ‘annotate’ command adds the version IRI to the ontology metadata. This OWL file is then converted to OBO format, at which point all targets are copied to a dated release directory.

### The Ontology Development Kit

Creating a Makefile to coordinate all these steps can be challenging. We make this easier for ontology developers by providing an Ontology Development Kit (ODK) [35]. This can be used to create a GitHub repository following a standard layout, with a standard Makefile following the workflow detailed above. The resulting GitHub repository will also be automatically configured to run the validation steps (such as ‘report’) of the workflow via Travis CI [21]. The workflow can also be executed using Docker with ODK containers released on Dockerhub [36]. This allows easy execution of workflows on either the local computer of an ontology developer, with Travis CI, or through scalable-build tools such as Jenkins [37].

ODK builds on ROBOT and demonstrates ROBOT’s utility, but a full discussion is beyond the scope of this article.

## Results and discussion

While there are many other Semantic Web and OWL development tools available, a number of factors have driven the OBO community to build and support ROBOT. First, the OBO commitment to open source development is a strong reason to use open source software. Second, our wide reliance on the free and open Protégé editor is a strong reason to use the same OWL API library that it is built upon. Third, there is a strong incentive to pool our limited resources and invest in shared tooling. Fourth, the OBO community has a range of conventions that support interoperability, and our workflows are simpler if we build these assumptions into the tools. Points three and four are clearly in tension: what is the right balance to strike between reusing general Semantic Web tools and building our own? Protégé, OWL API, and various OWL reasoners are general tools that we use, for instance, but we have strong conventions in our community for identifiers, release artifacts, metadata, quality control, etc. that these tools do not help us to enforce. Our compromise is to reuse open source tools as much as practical and invest community resources in customizing general tools such as OWL API to serve the needs of our community. A growing number of ontology developers are using ROBOT to help automate their quality-checking and release workflows. Two case studies are described here.

### Ontology for Biomedical Investigations

The Ontology for Biomedical Investigations (OBI) is an OBO Foundry ontology that aims to describe the processes, agents, devices, inputs, and outputs of scientific investigations [38]. When the project began more than a decade ago, development was done in Protégé, without any automation, and hosted on SourceForge. Today, OBI uses ROBOT to implement an automated workflow, supported by GitHub pull requests and Travis CI testing. More than 50 people have contributed to OBI development, including two of the authors of this paper (Overton and Jackson).

OBI has always imported a range of terms from other OBO projects, and OBI developers have maintained a number of separate OWL files to facilitate concurrent development by different groups of developers. When it comes time to prepare a new release of OBI, the various OWL files must be merged, tested and reasoned over.

In the early days of the project, OBI developer Alan Ruttenberg wrote a series of scripts for quality control and common operations, but merging, reasoning, and testing a new release still involved many hours of work by OBI developers. In 2013, James Overton developed a precursor to ROBOT: an automated build tool written in Java, using OWL API and Apache Ant, that automated some of the build, test, and release processes. This drastically reduced the time required to make a release, allowing for more frequent releases. While this code was specific to OBI workflows, some of it was used in early versions of ROBOT.

In 2017 OBI moved from SourceForge to GitHub and the release workflow was updated to use ROBOT throughout. This change vastly increased the degree of automation for ontology development tasks, expanded capabilities, and allowed OBI to pool some of its development resources with the wider OBO community to support shared tooling. OBI currently uses a Makefile [39] that defines a range of tasks for managing imports, converting templates, merging, reasoning, testing, and releasing new versions of OBI. The Makefile specifies various target files, and most target files are generated from a single ROBOT command or a single chain of ROBOT commands. The key steps are:

1. Update imports from upstream ontologies (currently using Ontofox [6]). OBI imports subsets of terms from more than a dozen OBO projects. As discussed, ROBOT supports this functionality with ‘extract’, but OBI’s use of Ontofox predates ROBOT development and has not yet been migrated.
2. Normalize RDF/XML for cleaner history of changes in the version control system (‘robot convert’). Different versions of OWL API have slightly different serialization behavior, which can lead to spurious reports of differences that make it more difficult to see relevant changes to the source files.
3. Convert template files (TSV) to OWL modules (‘robot template’). Templates often make it easier for domain experts to contribute to ontology development and enforce ontology design patterns that improve the overall quality of OBI.
4. Merge imports and templates with ‘obi-edit.owl’ (‘robot merge’). OBI uses a number of import and template files to enforce a separation of concerns, rather than making all changes in a single source file. These are merged into a single release file.
5. Use ‘SPARQL CONSTRUCT’ queries to update various term annotations (‘robot query’). Some standard term metadata can be automatically added and updated, rather than manually maintained.
6. Run an automated test suite (‘robot verify’). A range of quality control checks helps to ensure that errors have not been introduced into OBI by recent changes.
7. Run the HermiT reasoner (‘robot reason’). Reasoning checks the logical consistency of OBI and performs automated classification of terms.
8. Update annotations for release (‘robot annotate’). These annotations include the dated version IRI of this release of OBI.
9. Extract the OBI Core subset (‘robot extract’). The OBI Core subset provides approximately 100 important terms for educational and documentation purposes.
10. Create a list of OBI terms (‘robot query’). The term list is used to report on the new terms added to OBI with each release.

### Disease Ontology

The Disease Ontology (DO) is an OBO Foundry ontology that provides a standardized description of human diseases, including the phenotypic characteristics, symptoms, genetic bases, and related medical terminology. These terms are used by various model organism databases to provide a consistent representation of diseases [40]. The DO is developed at the University of Maryland School of Medicine by Lynn Schriml and her team, which currently includes one of the authors (Jackson).

In order to accurately and thoroughly describe the different aspects of diseases, DO makes use of more than 10 other biological ontologies. In the past, all DO imports were manually created and maintained. This led to inconsistencies as ontologies were updated and expanded, and also made it very difficult to add new entities to the imports.

In 2018, DO switched their entire automated build process (contained in the Makefile) from OWLTools [14] and OORT [15] to ROBOT. Instead of manually updating import modules, all required entities are now specified in text files. When a developer wishes to add a new imported entity, they simply add a line to the text file and run ‘make imports’. All imports are automatically regenerated during releases, as well, to keep the information up-to-date.

Before ROBOT, the monthly DO releases took multiple hours to run and required additional hours of manual editing and review. Now, DO developers simply run the ‘make release’ command and all content is generated in less than 20 min. The release process makes use of ROBOT commands such as ‘report’ to run quality control checks over the release products and reduce the time spent reviewing output.

Both ROBOT ‘verify’ and ‘report’ are used for DO’s Travis CI system [21]. Each time a new commit is pushed to the GitHub repository, a series of queries is run against the ontology files to ensure they meet certain standards. If they do not (or if ‘report’ fails with an ERROR-level violation), Travis CI notifies developers that the checks have failed with a red “X” next to the commit. Clicking on the red “X” will take the user to Travis CI, where they can see the command log to determine what caused the failure. On success, a green checkmark is displayed next to the commit to show that the checks have passed.

## Conclusions

ROBOT makes it easy for ontology developers to automate a wide range of tedious and error-prone development tasks, freeing their time to focus on other important parts of the ontology life cycle. Circa 2010, most OBO projects were manually edited, with manual imports, manually tested, and manually released using only Protégé. With ROBOT, ontology developers have a convenient tool for building powerful workflows that include format conversion, reasoning, extracting, querying, updating, testing, reporting, templating, and more. Low-level OWL API and Apache Jena operations are packaged into a library of high-level operations, and these operations are wrapped in a convenient command-line interface that is supported on the common server and desktop platforms. With ODK, developers benefit not only from ROBOT, but additionally from a wide range of best practices and standard procedures developed and shared by the OBO community.

ROBOT is open source software developed by a community of a dozen contributors with more than 1000 commits, hundreds of closed issues, and six releases. The ROBOT source code is freely available on GitHub and Maven Central. Documentation for the library is available on Javadoc.io and documentation for the commands is available on our website at http://robot.obolibrary.org, where you will also find examples of usage, test files, and explanations of common errors.

With ROBOT, we have tried to strike a balance between general tools such as Protégé and the specific needs of the OBO community, and to deliver the benefits of automation from software development to ontology development.

### Availability and requirements

**Project name:** ROBOT (ROBOT is an OBO Tool).

**Project home page:** http://robot.obolibrary.org/

**Operating system(s):** Platform-independent.

**Programming language:** Java 8.

**Other requirements:** None for the command-line tool. The ROBOT library depends on the following: Apache Jena, SnakeYAML, OpenCSV, FasterXML Jackson, OWL API, Apache Commons IO, Apache Maven, JSONLD-JAVA, Protégé, JUnit, SLF4J, and fmt-maven-plugin.

**License:** ROBOT is available under BSD 3. Dependencies are available under Apache 2.0 (Jena, SnakeYAML, OpenCSV, Jackson, OWL API, Commons IO, and Maven), BSD 3 (JSONLD-JAVA and Protégé), EPL-1.0 (JUnit), and MIT License (SLF4J and fmt-maven-plugin).

**Any restrictions to use by non-academics:** None other than those specified by the licenses.

## Data Availability

All code is available from https://github.com/ontodev/robot/

## Abbreviations

CSV: Comma-separated values
DL: description logic
DO: Disease Ontology
DOS-DP: Dead Simple OWL Design Patterns
EMR: Expression Materializing Reasoner
GUI: graphical user interface
OBI: Ontology for Biomedical Investigations
OBO: Open Biological Ontologies
ODK: Ontology Development Kit
OWL: Web Ontology Language
ROBOT: ROBOT is an OBO Tool
TSV: Tab-separated values
